# *Lactobacillus paracasei* JY062 Alleviates Glucolipid Metabolism Disorders via the Adipoinsular Axis and Gut Microbiota

**DOI:** 10.3390/nu16020267

**Published:** 2024-01-16

**Authors:** Yue Su, Jing Ren, Jingwen Zhang, Jiapeng Zheng, Qi Zhang, Yueling Tian, Yu Zhang, Yujun Jiang, Wei Zhang

**Affiliations:** Key Laboratory of Dairy Science, Ministry of Education, Department of Food Science, Northeast Agricultural University, Harbin 150030, China; 15604557325@163.com (Y.S.); renjing1979@126.com (J.R.); 13212939963@163.com (J.Z.); 18800468725@163.com (J.Z.); 18846833582@163.com (Q.Z.); tianyueling2022@163.com (Y.T.); jessedevil@163.com (Y.Z.); yujun_jiang@163.com (Y.J.)

**Keywords:** *Lactobacillus paracasei* JY062, glucolipid metabolism disorder, adipoinsular axis, gut microbiota

## Abstract

Glycolipid metabolic disorders (GLMD) refer to a series of metabolic disorders caused by abnormal processes of glucose and lipid synthesis, decomposition, and absorption in the body, leading to glucose and lipid excess, insulin resistance, and obesity. Probiotic intervention is a new strategy to alleviate metabolic syndrome. *Lactobacillus paracasei* JY062 (*L. paracasei* JY062) was separated from the Tibet-fermented dairy products. The results demonstrated a strong ability to relieve blood glucose disorders, blood lipid disorders, and tissue damage. The LPH group had the best effect, significantly decreasing the total cholesterol (TC), triglycerides (TG), low-density lipoprotein cholesterol (LDL-C), leptin, insulin, and free fatty acid (FFA) concentrations and increasing the high-density lipoprotein cholesterol, adiponectin, and *GLP-1* level compared to HFD-group mice. *L. paracasei* JY062 could activate the *APN-AMPK* pathway, increased *AdipoQ, AMPK GLUT-4*, and *PGC-1α* mRNA expression and decreased *SREBP-1c*, *ACC*, and *FAS* mRNA expression. *L. paracasei* JY062 intervention decreased the relative abundance of harmful bacteria, increased the relative abundance of beneficial bacteria, and restored the imbalance of gut microbiota homeostasis caused by a high-glucose-fat diet. *L. paracasei* JY062 alleviated glucolipid metabolism disorders via the adipoinsular axis and gut microbiota. This study provided a theoretical basis for probiotics to ameliorate glucolipid metabolism disorders by regulating the adipoinsular axis.

## 1. Introduction

In recent years, disorders of glucose and lipid metabolism have gradually become global epidemic diseases, mainly including hyperlipidemia, diabetes, and other diseases, and seriously threatening human health [1]. Some researchers have found that disorders of glucose and lipid metabolism are caused by a variety of factors, such as the abnormal metabolic processes of glucose and lipid synthesis and decomposition and absorption in the body, which lead to excess lipids, insulin resistance, and liver damage [2]. It is worth noting that dietary intervention is considered to be an effective and safe method for regulating metabolic pathways and reducing risk [3], which makes the demand for safer and more-effective natural health foods more urgent than ever.

Probiotics are defined as microorganisms that can exert health benefits for the host [4]. Among the recognized probiotics, lactic acid bacteria were the most frequently used probiotics in humans [4] due to their health-promoting properties, such as regulating immune function [5,6], reducing blood lipids, and regulating gut microbiota balance [7], anti-oxidation, and hypoglycemia [8], and their effectiveness has been demonstrated in various in vitro and in vivo studies [9]. Some studies have also shown that lactic acid bacteria strains were able to alleviate obesity in mice induced by a high-fat diet by regulating the content of leptin and adiponectin to relieve fat accumulation and lipid metabolism [10]. In our previous study, *Lactobacillus paracasei* JY062 (*L. paracasei* JY062) was separated from the Tibet-fermented dairy products. Our previous studies have demonstrated that *L. paracasei* JY062 had high α-glucosidase inhibitory activity and anti-diabetes ability and could survive in simulated gastrointestinal fluids. In addition, in diabetic mice induced by a combination of STZ and high-glucose-fat diets, the administration of *L. paracasei* JY062 can improve lipid metabolism, oxidative stress, and glucose metabolism abnormalities [11]. These results suggested that *L. paracasei* JY062 played an important role in the prevention of type 2 diabetes.

There is an adipoinsular axis in the human body. Insulin can promote the secretion of leptin, adiponectin, and other adipocytokines. The synthesis and secretion of insulin can also be directly suppressed, reversing high insulinmia and thus correcting the abnormalities of glycolipid metabolism [12]. Some studies have shown that active substances such as Tetradecyl 2,3-Dihydroxybenzoate can balance the adipoinsular axis to increase insulin and leptin generation and secretion and increase the sensitivity of insulin and leptin, thereby correcting the abnormalities of glycolipid metabolism [13]. However, the specific mechanism by which the adipocytokines and insulin regulate the metabolism of the glycolipid through the adipoinsular axis remains unclear.

Although *L. paracasei* JY062 was confirmed to correct glucose metabolism in diabetic mice, whether *L. paracasei* JY062 could improve glycolipid metabolism disorders based on the adipoinsular axis remains to be studied. The purpose of this study was to evaluate the effect of *L. paracasei* JY062 on glucolipid metabolism disorders induced by high-glucose-fat diets in mice and to explore the possible mechanism of *L. paracasei* JY062 in improving glucolipid metabolism disorders based on the adipoinsular axis.

## 2. Materials and Methods

### 2.1. Strain and Culture

*L. paracasei* JY062 was previously isolated from fermented dairy products in Tibet, China, and stored at −80 °C until use. Genome sequencing of the strain has been uploaded to NCBI. *L. paracasei* JY062 was inoculated into MRS liquid medium (Qingdao Haibo Co., Qingdao, China) the experiment. The oral bacterial solution samples were centrifuged at 4 °C 8000 r/min for 10 min, and cell microspheres were collected. After washing twice, they were suspended in 0.01 M PBS buffer (pH 7.4) at concentrations of 109, 108, and 107 CFU/mL for further experiments.

### 2.2. Animal Groups and Feeding

**Figure 1:**
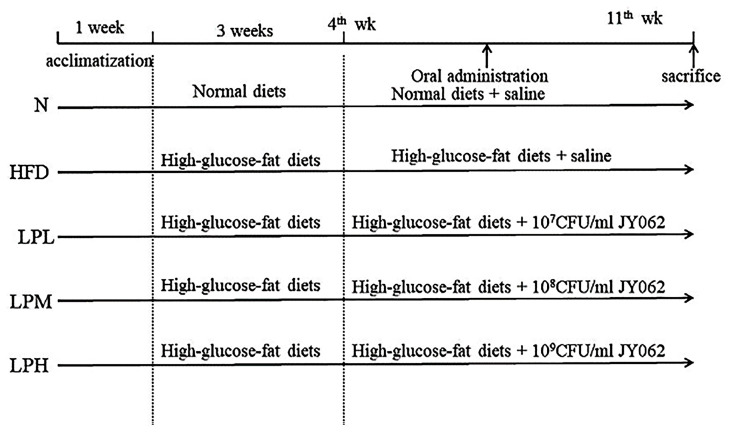
The flowchart of experimental protocol.

Fifty-five C57BL/6J male mice (18 ± 2 g, 4-week-old) were purchased from Weitong Lihua Laboratory Animal Technology Co., Ltd. (Beijing, China). All animal procedures were performed in accordance with the Guidelines for Care and Use of Laboratory Animals of Northeast Agricultural University, and experiments were approved by the Animal Ethics Committee of Northeast Agricultural. All mice were housed at 22 ± 2 °C and 55 ± 10% relative humidity and were exposed to a photoperiod of 12 h light and 12 h dark. After 7 days of administration with normal diet, the mice were randomly divided into 5 groups (11 mice in each group): normal group (N), model group (HFD), high-dose *L. paracasei* JY062 group (LPH), medium-dose *L. paracasei* JY062 group (LPM), and low-dose *L. paracasei* JY062 group (LPL). The bacterial suspensions of 10^9^ CFU/kg bw, 10^8^ CFU/kg bw and 10^7^ CFU/kg bw in LPH, LPM, and LPL groups were administered by gavage at a dose of 0.2 mL [14], and the same dose of normal saline was intragastrically administered in N and HFD groups once a day. The specific intervention of mice in each group was shown in Figure 1.

### 2.3. Sample Collection

The experimental mice were fed for 11 weeks. On the last day of the experiment, orbital blood was taken from the mice after fasting for 12 h; then, the serum was separated then stored at −20 °C for later use. Body weight was recorded every week. The mice were sacrificed, the abdominal cavity was opened, and the cecum and colon were cut off, and their contents were placed in 1.5 mL RNA free centrifuge tubes. The livers and pancreas were quickly removed from mice, washed with chilled normal saline, dried with filter paper, weighed, then stored at −80 °C until use.

### 2.4. Oral Glucose Tolerance Test (OGTT)

After 11 weeks of feeding, mice were subjected to 12 h of fasting and then orally administered a glucose bolus (2 g/kg body weight). Blood was drawn from the tail vein at 0, 30, 60, and 120 min after bolus administration. Glucose levels were measured using a blood glucose meter (Roche Diagnostics, Filderstadt, Germany), and the area under the curve (AUC) of blood glucose concentration (0–120 min) was calculated.

### 2.5. Serum Biochemistry Determination

The collected mice blood was placed at room temperature for 2 h then centrifuged at 4 °C, 3000 r/min for 10 min, and the serum was collected. Enzyme-linked immunosorbent assay (ELISA) kits were used to measure TC, TG, HDL-C, and LDL-C.

### 2.6. Determination of Adipokines and Insulin on the Fat–Adipoinsular Axis

According to the instructions of relevant ELISA kits, the contents of Adiponectin (ADPN), leptin (LEP), free fatty acid (FFA), insulin (INS), and Glucagon-like peptide-1 (GLP-1) on the fat–adipoinsular axis of mice in each group were measured.

### 2.7. Liver and Pancreas Histological Analysis

The liver and pancreas tissues fixed in 4% paraformaldehyde solution were embedded in paraffin, sectionalized, and stained with Hematoxylin and eosin stain (H&E). Finally, the histopathological changes of liver and pancreas were observed and photographed by a 400× light microscope.

### 2.8. Immunofluorescence Analysis of Insulin in Mouse Pancreas

Pancreatic tissue samples were deparaffinized and placed in antigen repair buffer at pH 8 for antigen repair and serum closure. After adding primary antibody, secondary antibody, and autofluorescence quencher, the reaction was carried out for 5 min and washed with water for 10 min. The nuclei of the cells were restained using DAPI, and the nuclei were closed by applying antifluorescence-quenching sealant. After the slices were sealed, the slices were photographed by placing them under the observation of a fluorescence microscope, and the area of the pancreatic sclerotium was calculated.

### 2.9. Gene Expression Analysis

The cycling conditions and calculation methods of RT-PCR was carried out according to previous method [15]. Primer 5.0 software was used to design specific primers, and primer synthesis was performed by Shanghai Sangyo Biologicals Co. Total RNA from mice livers was extracted. Reverse transcription of RNA into cDNA was carried out according to the kit operating instructions. The primers used in the experiment are listed in Table 1.

### 2.10. Western Blot Assay

The specific steps of the Western blot test were adopted and improved from the method of Shi et al. [16], which adjusts exposure conditions for different light intensities and develops and fixes film. The film is scanned and archived, and the Image-Pro Plus 6.0 software processing system analyzes the optical density values of the target strip.

### 2.11. Analysis of Gut Microbiota Composition

DNA from mice fecal samples was extracted using a fecal DNA extraction kit. The Illumina MiSeq sequencer was used and performed to detect the composition/proportion of the main bacterial groups in the fecal samples. Multivariate statistical analysis, such as principal component analysis (PCA) and orthogonal partial least squares discriminant analysis (OPLS-DA), was carried out.

### 2.12. SCFAs Analysis

The feces of the mice were collected and stored at −80 °C. Then, the mice feces were freeze-dried and weighed. The content of SCFAs in feces was determined according to the previous method [17].

### 2.13. Statistical Analysis

One-way analysis of variance (ANOVA) was performed by SPSS 23 and Origin 2021 software. Graph production was performed by Excel and GraphPad Prism 8.02. Experimental results were repeated three times and expressed as mean ± standard deviation (mean ± SD), and *p* < 0.05 indicated a significant difference. Multivariate statistical analysis, such as principal component analysis (PCA) and orthogonal partial least squares discriminant analysis (OPLS-DA), were carried out.

## 3. Results

### 3.1. Changes of Body Weight and Blood Glucose in Mice

**Figure 2:**
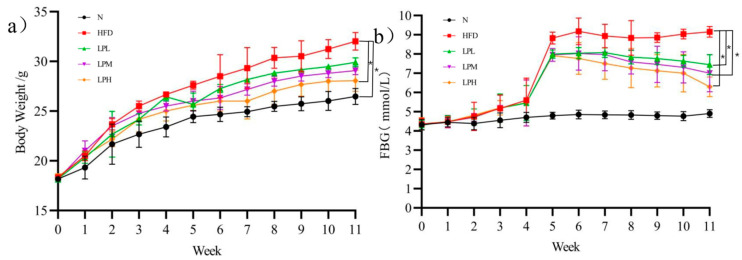
Effects of *L. paracasei* JY062 on body weight (**a**) and FBG (**b**) of mice (*n* = 11). The “*” denotes a significant difference between different groups of mice (*p* < 0.05).

The body weight changes of mice were shown in Figure 2a. During the experiment, mice in N group gained weight slowly. Within 4 weeks of the experiment, the weight of the mice fed the high-glucose-fat diet increased continuously and was always higher than that of the mice fed the normal diet. At the fourth week, the mice fed the high-glucose-fat diet had a 45.5%-higher body weight gain than those in the N group (23.4 ± 1.01 g). After the fifth week, the body weight growth rate of mice in the LPL, LPM, and LPH groups was slower, and the body weight gain was significantly lower than that in HFD group. At the end of the experiment, the body weight of mice in the LPH group (28.07 ± 1.07 g) was significantly lower than the body weight of mice in the HFD group (32.03 ± 0.89 g) (*p* < 0.05).

The changes of fasting blood glucose in mice are shown in Figure 2b. During the experiment, the changes of blood glucose in group N were not obvious. Within 4 weeks of the experiment, there was no significant difference in the blood glucose of mice fed a high-glucose-fat diet compared with the N group, but the blood glucose began to increase significantly in the fifth week up to 8.83 mmol/L. After the administration of *L. paracasei* JY062, the blood glucose of mice could decrease significantly. At the end of the experiment, the blood glucose in the LPL, LPM, and LPH group was 7.44, 7.00, and 6.29 mmol/L, respectively, significantly lower than that in the HFD group 9.17 mmol/L (*p* < 0.05). Therefore, *L. paracasei* JY062 could alleviate the changes of body weight and blood glucose with glycolipid metabolic disorder, thereby improving the symptoms of glycolipid metabolic disorder.

### 3.2. Changes of Oral Glucose Tolerance in Mice

**Figure 3:**
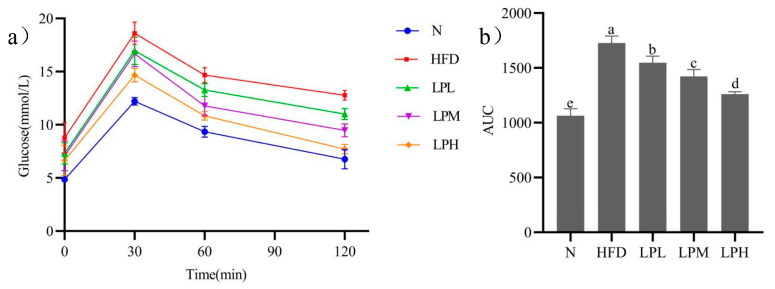
Effects of *L. paracasei* JY062 on Glucose (**a**) and AUC (**b**) of mice (*n* = 11). Different letters denote a significant difference between different groups of mice (*p* < 0.05).

The changes of oral glucose tolerance in mice were shown in Figure 3a. Due to the administration of glucose, the blood glucose concentration of the mice increased rapidly within 30 min (N group: 12.2 ± 0.36 mmol/L; HFD group: 18.6 ± 1.05 mmol/L; LPL group: 16.9 ± 1.29 mmol/L; LPM group: 16.6 ± 1.21 mmol/L; LPH group: 14.7 ± 0.64 mmol/L), but the blood glucose concentration of mice decreased significantly at 1 h (N group: 9.3 ± 0.51 mmol/L; HFD group: 14.7 ± 0.71 mmol/L; LPL group: 13.3 ± 0.6 mmol/L; LPM group: 11.8 ± 0.91 mmol/L; LPH group: 10.8 ± 0.4 mmol/L), and at 2 h, the blood glucose concentration of mice in the N group returned to a normal level, the blood glucose concentration of mice in the HFD group was still at a high level (12.6 ± 0.72 mmol/L), and the blood glucose of mice in the LPL, LPM, LPH groups decreased significantly compared to the HFD group but did not return to normal levels, which were 11.1 ± 0.61 mmol/L, 9.5 ± 0.7 mmol/L, and 7.7 ± 0.5 mmol/L, respectively.

Glucose tolerance of mice was shown in Figure 3b. The AUC glucose value of the HFD, LPL, and LPM LPH groups were significantly higher than that of the N-group mice. Compared with the HFD group (1728), the AUC glucose value of LPL and LPM LPH groups was significantly decreased (*p* < 0.05), and the AUC glucose value of LPH group was the lowest (1260). The results showed that *L. paracasei* JY062 could improve glucose tolerance in a dose-dependent manner.

### 3.3. Changes of Blood Lipid Index in Mice

The changes in the blood lipid index in mice are shown in Table 2. After 10 weeks of high-glucose-fat diet, the mice in the HFD group significantly (*p* < 0.05) increased the TC (1.09 times), TG (84.5%), and LDL-C (1.08 times) concentrations and decreased (*p* < 0.05) the HDL-C (50.27%) level compared to N-group mice. After 7 weeks of *L. paracasei* JY062 intervention, TG, TC, and LDL-C in different dose groups of *L. paracasei* JY062 were significantly decreased compared to the HFD group, while HDL-C was significantly increased (*p* < 0.05). The LPH group had the best effect, significantly (*p* < 0.05) decreasing the TC (40.11%), TG (42.46%), and LDL-C (39.38%) concentrations and increasing (*p* < 0.05) the HDL-C (1.05 times) level compared to HFD-group mice.

### 3.4. Changes on the Adipoinsular Axis

**Figure 4:**
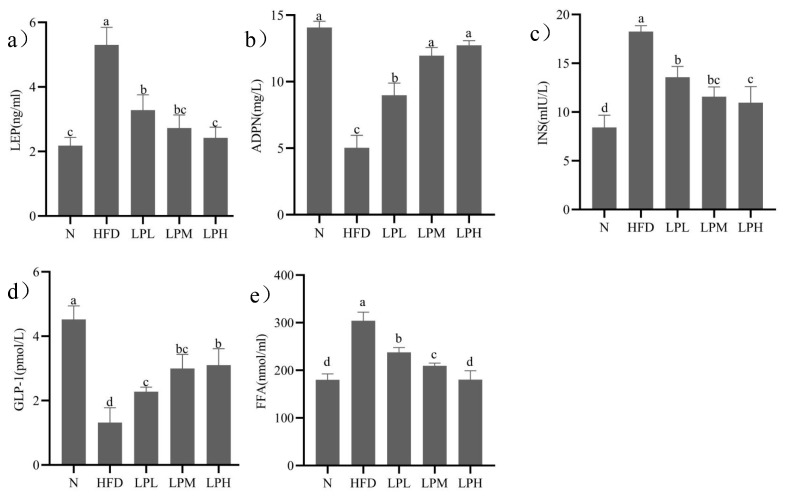
Effects of *L. paracasei* JY062 on leptin (**a**), adiponectin (**b**), insulin (**c**), GLP-1 (**d**), and FFA (**e**) of mice (*n* = 11). Different letters denote the significant difference between different groups of mice (*p* < 0.05).

The changes of leptin, adiponectin, insulin, GLP-1, and FFA in mice were shown in Figure 4.

After 3 weeks on a high-glucose-fat diet, the mice in the HFD group significantly (*p* < 0.05) increased their leptin (1.43 times), insulin (1.17 times), and FFA (68.80%) concentrations and decreased their adiponectin (64.25%) and GLP-1 (71.02%) level compared to N-group mice. After 7 weeks of *L. paracasei* JY062 intervention, compared with the HFD group, the contents of leptin, insulin, and FFA in different dose groups of *L. paracasei* JY062 were significantly decreased, while the contents of adiponectin and GLP-1 were significantly increased. LPH group had the best effect, significantly (*p* < 0.05) decreasing the leptin (54.34%), insulin (39.95%), and FFA (40.68%) concentrations and increasing the adiponectin (1.53 times) and GLP-1 (1.35 times) level compared to HFD-group mice.

### 3.5. Histopathological Analysis of Liver and Pancreas

**Figure 5:**
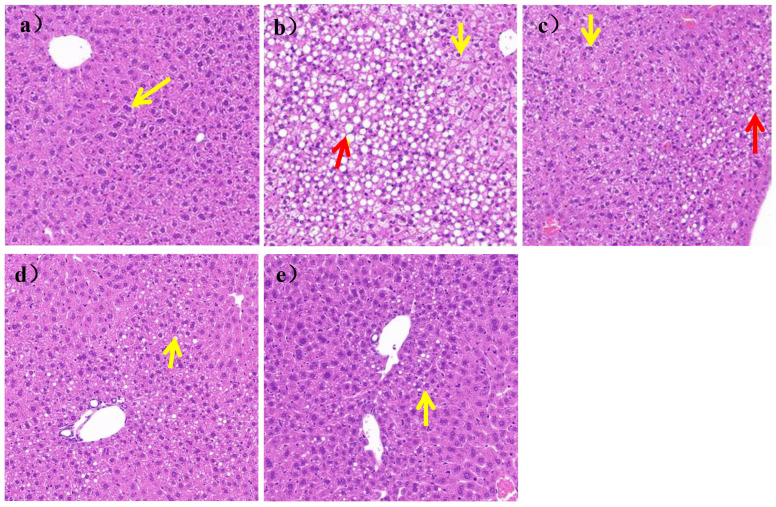
Image of liver section (scale bar = 50 μm). (**a**) mice liver of N group; (**b**) mice liver of HFD group; (**c**) mice liver of LPL group; (**d**) mice liver of LPM group; (**e**) mice liver of LPH group; The yellow arrows represent hepatocyte edema; The red arrows represent fat degeneration.

As shown in Figure 5a, the liver structure of mice in the N group was mildly abnormal, with mild edema of some liver cells, clear nuclei, and vacuolated cytoplasm (yellow arrows). There was no inflammatory cell infiltration and steatosis in liver parenchyma of the N group, while, as shown in Figure 5b, the liver structure of the mice was severely abnormal, with a large area of hepatic cell steatosis and a large number of lipid droplets of different sizes in the HFD group (yellow arrows). Some cells were severely edematous, swollen, and vacuolized, as shown by the red arrow in Figure 5b.

**Figure 6:**
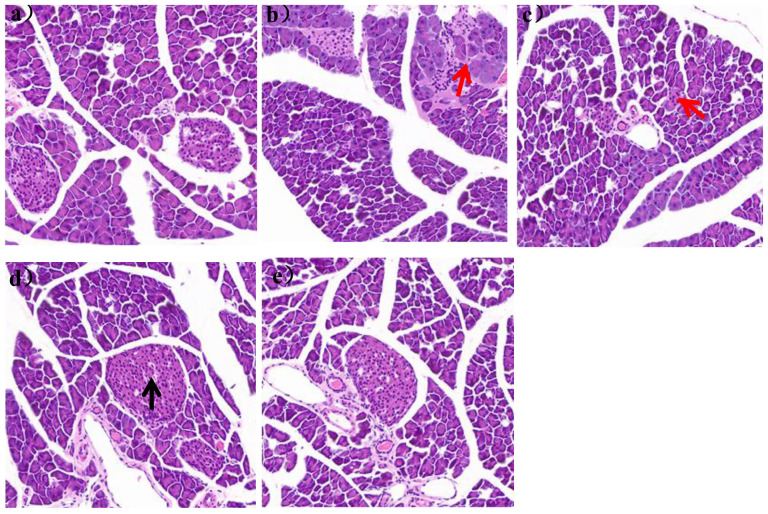
Image of pancreatic section (scale bar = 50 μm). (**a**) mice pancreas of N group; (**b**) mice pancreas of HFD group; (**c**) mice pancreas of LPL group; (**d**) mice pancreas of LPM group; (**e**) mice pancreas of LPH group; The black arrows represent telangiectasia; The red arrows represent inflammatory cells.

After 7 weeks of *L. paracasei* JY062 intervention, compared with the HFD group, as shown in Figure 5c–e, the degree of edema and steatosis of liver cells was reduced, the liver cells were homogenized, and the liver cell space was reduced. In addition, among the three doses of *L. paracasei* JY062, the LPH group had the best effect, which an hepatocyte status was similar to that of the N group.;

It could be seen in Figure 6a that the pancreatic tissue structure of mice in group N was clear, without vacuolation and inflammatory cell infiltration.

The islet was a spherical cell-like structure, distributed between the acinus, with a clear boundary with the surrounding glands. And the islet cells were arranged regularly, with high cell density and abundant cytoplasm. In the HFD group, the islets were distributed between the acinus and the surrounding glands, and the islets were not clearly demarcated with the surrounding glands. The number of islets was also significantly reduced, with a large number of inflammatory cells infiltrating, as shown by the red arrow in Figure 6b. After 7 weeks of *L. paracasei* JY062 intervention, compared with the HFD group, the arrangement of islet cells gradually became regular, the number of islets gradually increased, and the boundary between islets and surrounding glands gradually became clear. In the LPH group, some telangiectations occurred, as shown by the black arrow in Figure 6d.

### 3.6. Immunofluorescence Analysis of Insulin in Mouse Pancreas

**Figure 7:**
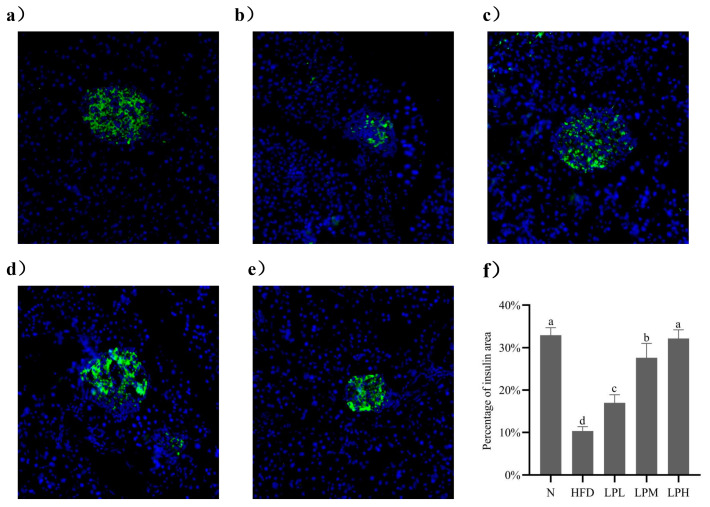
Immunofluorescence analysis of pancreatic tissue: (**a**) N group; (**b**) HFD group; (**c**) LPL group; (**d**) LPM group; (**e**) LPH group; (**f**) percentage of insulin area. Different letters denote the significant difference between different groups of mice (*p* < 0.05).

The occurrence of glucolipid metabolism disorders led to the abnormal secretion of insulin regulated by the body and then damage to the pancreatic tissue. Therefore, immunofluorescence technology was used to analyze the morphology of islets of mice in each group and calculate the area of insulin in the observation field. The area of insulin could reflect the ability of pancreatic tissue to secrete insulin. The larger the area, the stronger the ability to secrete insulin. As shown in Figure 7, insulin was labeled with green fluorescence, and the nucleus was labeled with blue fluorescence in pancreatic tissue.

As can be seen from the Figure 7, compared with the mice in the N group, in the HFD group, the islets labeled with green fluorescence were distributed between acini, the boundary with surrounding glands was not clear, and the islet area was significantly reduced by 22.19% (*p* < 0.05). This shown that the high-glucose-fat diet damaged the pancreas, which led to the islets’ abnormal β cell function. Although the remaining islet cells could still secrete insulin, the blood glucose level did not decrease, indicating that the mice had insulin resistance. Compared with the mice in the HFD group, after the intervention of *L. paracasei* JY062, we could observe that the arrangement of islet cells was gradually regular, that the boundary between islet cells and surrounding glands was gradually clear, and that the area percentage of insulin had a significant upward trend in a dose-dependent manner. Among them, the islet area percentage in the LPH group increased fastest, which was 25.17% higher than in the HFD group (*p* < 0.05). It was found that *L. paracasei* JY062 could alleviate pancreatic injury in mice with impaired glucolipid metabolism disorder, restore pancreatic cell morphology, and promote normal insulin secretion.

### 3.7. Effect of L. paracasei *JY062* on Gene Expression of Glycolipid Metabolism

**Figure 8:**
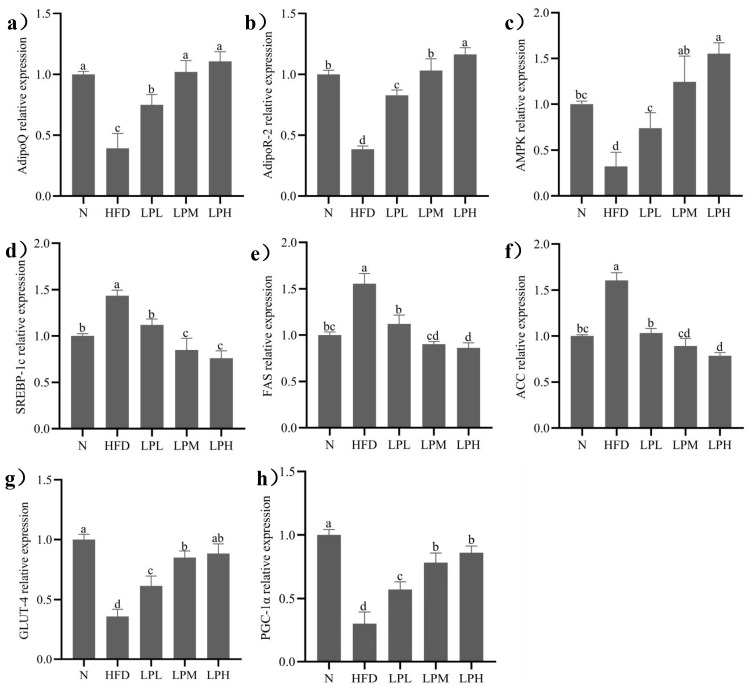
Effects of *L. paracasei* JY062 on mRNA expression of mice liver (*n* = 11). Different letters denote a significant difference between different groups of mice (*p* < 0.05). (**a**) the relative expression level of AdipoQ; (**b**) the relative expression level of AdipoR-2; (**c**) the relative expression level of AMPK; (**d**) the relative expression level of SREBP-1c; (**e**) the relative expression level of FAS; (**f**) the relative expression level of ACC; (**g**) the relative expression level of GLUT-4; (**h**) the relative expression level of PGC-1α.

The glycolipid metabolism-associated genes expression of experimental mice after diet intervention was shown in Figure 8. In the case of long-term intake of high-glucose-fat diet, *L. paracasei* JY062 relieved glycolipid metabolism disorders and reduced lipid accumulation and glycogen synthesis in mice. The expression levels of adiponectin-related genes (AdipoQ, Adipor2), AMPK, and insulin resistance-related genes (GLUT-4, PGC-1α) in the HFD group were significantly lower than in the N group (*p* < 0.05). And fat synthesis-related genes (SREBP-1c, ACC and FAS) in the HFD group were significantly higher than in the N group (*p* < 0.05).

**Figure 9:**
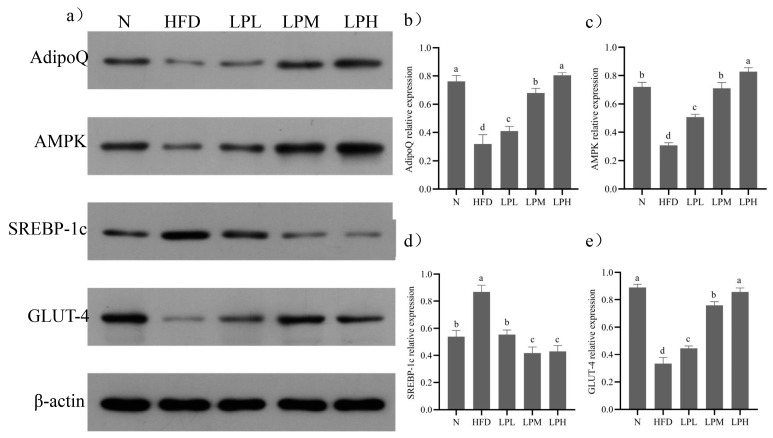
Effects of *L. paracasei* JY062 on protein expression of mice liver (*n* = 11). Different letters denote a significant difference between different groups of mice (*p* < 0.05). (**a**) the protein electrophoresis of AdipoQ, AMPK, SREBP-1c, and GLUT-4 in each group of mice (**b**) the protein relative expression level of AdipoQ; (**c**) the protein relative expression level of AMPK; (**d**) the protein relative expression level of SREBP-1c; (**e**) the protein relative expression level of GLUT-4.

After 7 weeks of *L. paracasei* JY062 intervention, compared with the HFD group, the gene expression levels of AdipoQ, Adipor2, AMPK, GLUT-4, and PGC-1α in different doses of *L. paracasei* JY062 group were significantly up-regulated (*p* < 0.05), and the gene expression levels of SREBP-1c, FAS, and ACC were significantly down-regulated (*p* < 0.05). Among them, the LPH group had the best effect, significantly (*p* < 0.05) increasing the AdipoQ (1.82 times), Adipor2 (2.02 times), AMPK (3.84 times), GLUT-4 (1.38 times), and PGC-1α (1.85 times) and decreasing (*p* < 0.05) the SREBP-1c (47.2%), FAS (44.6%), and ACC (50.9%) relative expression levels compared to HFD-group mice. Meanwhile, the key protein expressions in glycolipid metabolism are shown in Figure 9.

Compared with the N group, the protein expressions levels of AdipoQ, AMPK, and GLUT-4 in the liver tissue of the HFD group decreased (*p* < 0.05) and the protein expressions of SREBP-1c increased (*p* < 0.05). Compared with the HFD group, the expression levels of AdipoQ, AMPK, and GLUT-4 protein in the liver tissue of mice in the LPL, LPM and LPH groups increased (*p* < 0.05) and the protein expression levels of SREBP-1c decreased (*p* < 0.05). The LPH group had the best effect. Compared with the HFD group, the relative expression levels of AdipoQ, AMPK, and GLUT-4 were increased by 152%, 169%, and 157%, respectively. The relative expression of SREBP-1c protein decreased by 51.95% (*p* < 0.05). The results suggested that *L. paracasei* JY062 could activate adiponectin by regulating glycolipid disorder in mice with glycolipid metabolism disorder through the APN-AMPK pathway.

### 3.8. Gut Microbiota Analysis

**Figure 10:**
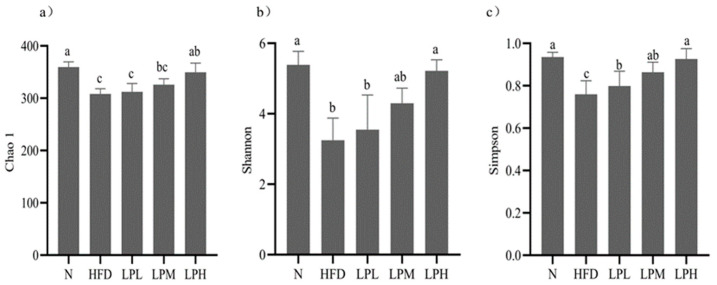
α diversity indexes of intestinal microbe species in mice. Different letters denote a significant difference between different groups of mice (*p* < 0.05). (**a**) Chao1 index; (**b**) Shannon index; (**c**) Simpson index.

As can be seen in the Figure 10, compared with the mice in the N group, the Chao1, Shannon, and Simpson indexes of the mice in the HFD group were significantly decreased after 10 weeks of feeding with a high-glucose-fat diet (*p* < 0.05).

These indicated that the richness and diversity of gut microbiota species of mice were significantly reduced when glucose and lipid metabolism disorders occurred. Compared with the HFD group, the LPL group had no significant difference in the Chao1, Shannon, and Simpson indexes, while the LPM group and the LPH group had significant differences (*p* < 0.05), and the LPH group had the best effects. The results suggested that *L. paracasei* JY062 can restore the richness and diversity of gut microbiota in mice with glucose and lipid metabolism disorders.

**Figure 11:**
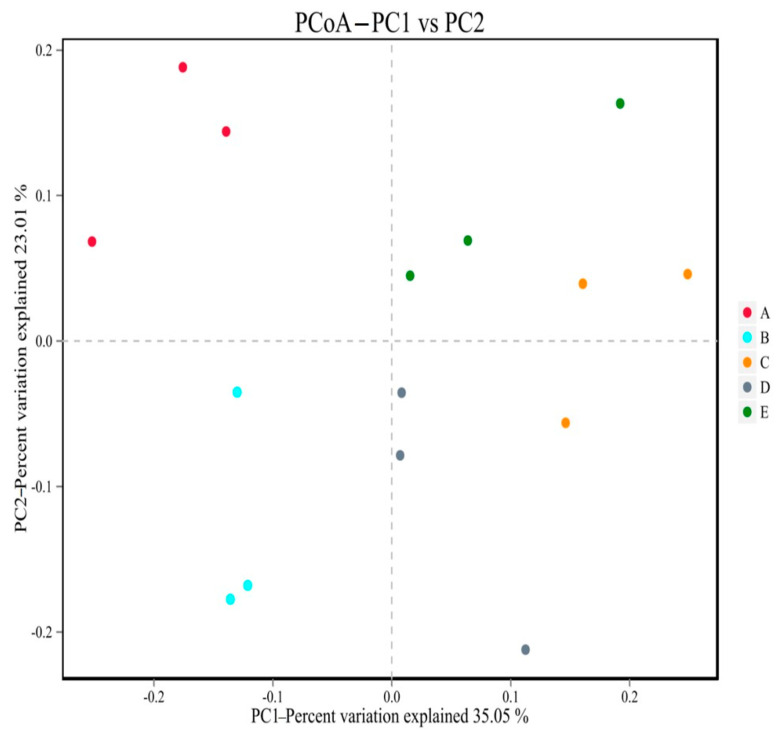
PCoA analysis of psychrotrophic microbiome in groups. A (N group); B (HFD group); C (LPL group); D (LPM group); E (LOH group).

The structure of gut microbiota of mice in group N was relatively similar, and the gut microbiota of mice in group N was relatively similar (Figure 11). The composition of flora species was relatively stable. The microbiota of HFD group mice was significantly different from that of the N group in the first and second principal coordinates, which showed that there were great changes in the gut microbiota of mice with glucose and lipid metabolism disorder. The LPL, LPM, and LPH groups were separated from the HFD group flora and gradually approached the N-group mice. The results also indicated that *L. paracasei* JY062 could regulate the gut microbiota structure of mice with glucose and lipid metabolism disorders to a certain extent and improved the similarity and stability of the gut microbiota community structure.

In this study, the relative abundances of Firmicutes and Bacteroidetes in the intestines of mice in the N group were 49.91% and 16.36%, the ratio of Bacteroidetes/Firmicutes was 0.328, and the relative abundances of Firmicutes and Bacteroidetes in the intestines of mice in the HFD group were 61.18% and 61.18%. The ratio of Bacteroidetes/Firmicutes, 17.11%, was 0.18, and the ratio of Bacteroidetes/Firmicutes decreased significantly compared with N group mice (*p* < 0.05). This showed that the ratio of Bacteroidetes/Firmicutes was significantly reduced in mice. In the LPL, LPM, and LPH groups, the ratios of Bacteroidetes/Firmicutes were 0.10, 0.14, and 0.15, respectively. In the LPH group, the relative abundances of Firmicutes and Bacteroidetes were 34.28% and 5.28%. The relative abundance decreased compared with the HFD group. In addition, Verrucomicrobia was proved to be closely related to the occurrence of diabetes and obesity, and the relative abundance of Verrucomicrobia was significantly increased in the LPH group, reaching 27.35% (*p* < 0.05). *L. paracasei* JY062 can improve glucose and lipid metabolism disorder to a certain extent by reducing the abundance of Firmicutes, increasing the abundance of Bacteroidetes and the relative abundance of Verrucomicrobia. However, the effect of probiotics on gut microbiota was very complicated.

**Figure 12:**
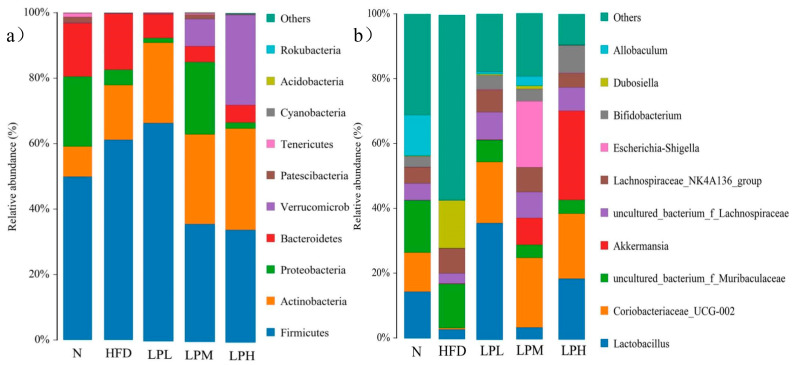
The microbial composition of mice intestinal flora at the phylum level and genus level. (**a**) Phylum level; (**b**) Genus level.

As could be seen from Figure 12, compared with the mice in the N group, the gut microbiota of the HFD group was dominated by *Dubosiella* (14.78%), *Lachnospiraceae NK4A136* group (7.66%), and uncultured *Muribaculaceae* bacterium (13.60%). Three harmful bacteria genera increased. *Lactobacillus* (3.07%), *Coriobacteriaceae*_UCG-002 (0.45%), *Akkermansia* (0.13%), *Bifidobacterium* (0.08%), and *Allobaculum* (0.01%) were the five main beneficial bacteria. The proportion had decreased. These results suggested that feeding a high-glucose-fat diet affects the composition of the intestinal microbiota in mice, thereby affecting the glucose and lipid metabolism in mice. In the LPL, LPM, and LPH groups, compared with the HFD group of mice, five genera, mainly *Lactobacillus*, *Coriobacteriaceae_UCG-002, Akkermansia, Bifidobacterium,* and *Allobaculum*, increased (*Dubosiella* by 14.78%, *Lachnospiraceae NK4A136* group by 7.66%). Four genera dominated by *Muribaculaceae* (13.60%) declined, among which, in the LPH group, *Lactobacillus* (18.77%), *Coriobacteriaceae_UCG-002* (19.92%), *Akkermansia* (27.35%), *Bifidobacterium* (8.5%) were the main dominant bacterial genera. *Lactobacillus*, *Coriobacteriaceae_UCG-002*, *Akkermansia*, and *Bifidobacterium* have protective effects in glucolipid metabolism diseases, such as obesity caused by diet. The above results indicated that *L. paracasei* JY062 could enhance the intestinal homeostasis of mice and increase the abundance of *Lactobacillus*, *Coriobacteriaceae_UCG-002*, *Akkermansia*, and *Bifidobacterium* to alleviate the abnormal glucose and lipid metabolism caused bya high-glucose-fat diet.

### 3.9. Effect of L. paracasei *JY062* on the Concentration of SCFAs

**Figure 13:**
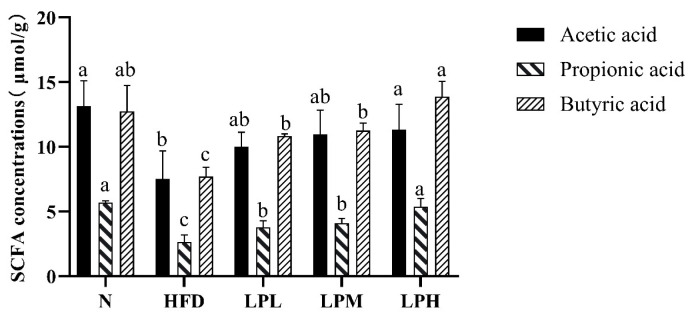
Effects of *L. paracasei* JY062 on intestinal SCFA concentrations in the mice feces (*n* = 11). Different letters denote a significant difference between different groups of mice (*p* < 0.05).

In order to further explore the mechanism of *L. paracasei JY062* in resisting glycolipid metabolism, the levels of SCFAs in the feces of each group of mice were analyzed, and the results are shown in Figure 13.

Among the SCFAs in the feces, compared with the N group, acetic acid, propionic acid, and butyric acid were significantly decreased in the HFD mice, which were reduced by 42.75%, 53.37%, and 39.56%, respectively. However, after 7 weeks of *L. paracasei* JY062 intervention, the concentration of acetic acid, propionic acid, and butyric acid were significantly increased, especially the concentration of butyric acid in the LPH group, which was 1.8 times higher than that of the HFD group (*p* < 0.05).

### 3.10. Relationship between SCFAs and Gut Microbiota

As the metabolites of gut microbiota, the content of SCFAs is closely related to the composition of gut microbiota. Lactobacillus and Bifidobacterium in the gut microbiota could stimulate the production of SCFAs. In this study, a heat map of the relationship between the content of acetic acid, propionic acid, and butyric acid in SCFAs and the composition of gut microbiota in mice was analyzed.

**Figure 14:**
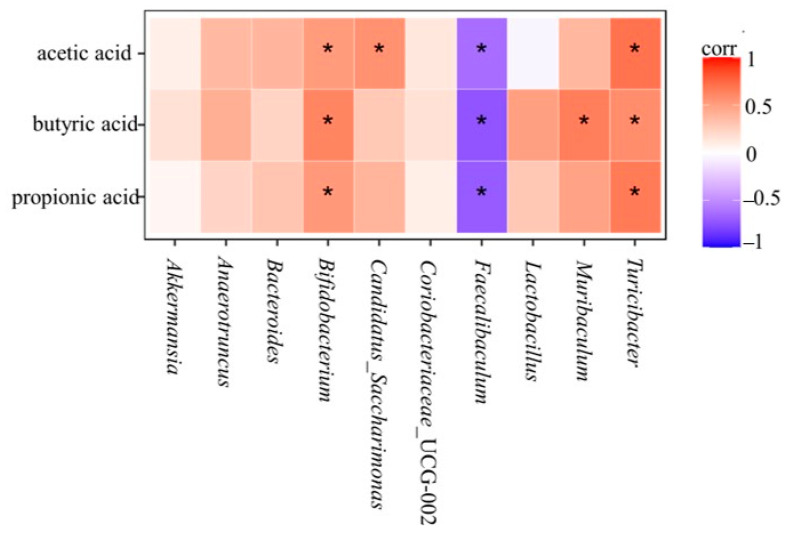
The Spearman correlation analysis between short-chain fatty acids and intestinal bacteria. The “*” denotes a significant difference between different groups of mice (*p* < 0.05).

As can be seen in Figure 14, the acetic acid content was positively correlated with Bifidobacterium, Candidatus_Saccharimonas, and Turicibacter and negatively correlated with Faecalibaculum. In the analysis of species composition at the genus level and the content of SCFAs, it was found that the abundance of Bifidobacterium decreased in the HFD group and the relative abundance of Faecalibaculum increased. The relative abundance of Candidatus_Saccharimonas and Turicibacter increased, and the relative abundance of Faecalibaculum decreased. This indicated that *L. paracasei* JY062 could increase acetate content by increasing the abundance of Candidatus_Saccharimonas and Turicibacter and decreasing the abundance of Faecalibaculum. In addition, butyric acid content was positively correlated with Bifidobacterium, Muribacalum, and Turicibacter and negatively correlated with Faecalibaculum. In the analysis of species composition at the genus level and the content of SCFAs, it was found that the relative abundance of Bifidobacterium decreased and the relative abundance of Faecalibaculum increased after a long-term high-sugar and high-fat diet.

## 4. Discussion

Originally, it was considered that obesity, especially abdominal obesity, was an important risk factor for insulin resistance. However, it has been found that fat atrophy can also lead to serious insulin resistance [18]. Some research results suggested that adipose tissue plays a very important role in regulating insulin sensitivity. Accordingly, probiotics have become an alternative intervention for glycolipid metabolism [19]. In recent years, the weight loss and anti-diabetes effects of probiotics and probiotics preparations have been confirmed in animal models and clinical trials [20]. Moreover, further evidence support that the gut microbiota, which can act on molecules that regulate appetite and satiety, plays a leveraging role in energy efficiency and adipose tissue accumulation [21]. Our results indicated that *L. paracasei* JY062 could alleviate high-glucose-fat-induced weight gain and fasting blood glucose elevation in mice.

Glycolipid metabolism disorder is a disease characterized by abnormal glucose and lipid metabolism. Therefore, we examined serum TC, TG, LDL-C, HDL-C, and OGTT. Long-term glycolipid metabolism disorder could bring about hepatic steatosis and liver injury characterized by abnormally elevated TC, TG, LDL-C, and lower HDL-C levels in liver, which caused damage to hepatocytes and glucose intolerance [22]. Therefore, successfully controlling serum cholesterol level was a powerful strategy to prevent the development of hyperlipidemia. Our results showed that *L. paracasei* JY062 intervention could ameliorate the hyperlipidemia and glucose intolerance induced by a high-glucose-fat diet, and *L. paracasei* JY062 has been shown to reduce the risk of obesity-related factors while lowering TC, TG, and LDL-C and increasing HDL-C. And our results were similar to Yang’s; *L. rhamnosus* JL1 intervene in mice on a high-fat diet for 10 weeks, which can effectively alleviate TG content in the mice serum [14].

Improvement of glucolipid metabolism disorders depends on adipokines and insulin on the adipoinsular axis. On the one hand, adipokines, including adiponectin and leptin, were adipocyte-derived products and associated with glucolipid metabolism, such as glucose, lipid, and energy homeostasis [23]. On the other hand, the protein that plays an important role in glucolipid metabolism is GLP-1, a glucose-dependent hormone secreted by intestinal endocrine cells. GLP-1 inhibited glucagon secretion and promotes insulin secretion when blood glucose was high [24]. Meanwhile, insulin can promote leptin secretion and inhibit lipolysis to improve insulin sensitivity, increase fat oxidation, and improve glucolipid metabolism [25]. In our study, it was found that the contents of leptin, insulin, and FFA in different dose groups of *L. paracasei* JY062 were significantly decreased, and the contents of adiponectin and GLP-1 were significantly increased (*p* < 0.05). Similar to our findings, administration of B. lactis BB-12 ameliorated glycolipid metabolism disorders by activating adiponectin and stimulating the secretion of intestinal hormone GLP-1 [26]. Taken together, these results explained that the adipoinsular axis played an important role in improving glycolipid metabolism disorder. The symptoms of glucolipid metabolism disorders are not only manifested in elevated blood glucose and lipid levels; they are also accompanied by pathological changes of pancreas, liver, and other organs [27]. The hepatic pathological sections illustrated that the liver cells in the HFD group had pathological conditions such as unclear boundary, disordered structure, enlarged cell space, and sparse distribution of liver glycogen. Pancreatic slices of mice in the model group showed pathological states such as changes in cell morphology and decreased number, which were similar to the results of Zheng [28]. *L. paracasei* JY062 intervention significantly improved the liver and pancreas health status. Our result was consistent with Yan’s [29]. In the present study, we found that after the intervention of *L. paracasei* JY062, mice showed a significant decrease in leptin, insulin, and FFA levels, a significant increase in the islet area in pancreatic immunofluorescence maps, and a significant increase in both lipocalin and GLP-1 levels (*p* < 0.05) in a dose-dependent manner. This suggests that *L. paracasei* JY062 can improve leptin and insulin resistance in GLMD mice by increasing the levels of lipocalin and GLP-1 and decreasing the leptin level, which, in turn, improves the disorders of glucolipid metabolism.

Liver is an important metabolic organ. It plays a critical role in glucolipid metabolism [30]. To elucidate the possible mechanisms of *L. paracasei* JY062 in the improvement of glucolipid metabolism disorders, we measured the expression of genes related to glycogenesis, lipogenesis, and the insulin signaling pathway. AdipoQ, derived from adipocytes, and can bind to adiponectin receptors 1 and 2, thereby enhancing insulin sensitivity [31]. In the liver, AdipoR2 activates adenosine-activated protein kinase AMPK. In the APN-AMPK pathway, AMPK was the main energy sensor in cells of the body and involved in fatty acid oxidation, glucose transport, triglyceride synthesis and metabolism, and other cellular metabolic processes. It was also an important downstream factor of the APN signaling pathway [32]. Activated AMPK inhibits the activity of SREBP-1C, which was an important regulator of lipid synthesis and regulated adipocyte differentiation and intracellular ectopic accumulation of fat [33]. Downstream target genes of SREBP-1C were ACC and FAS. FAS was the key enzyme of endogenous fatty acid synthesis, and ACC was the rate-limiting enzyme of fatty acid synthesis, which was closely related to the rate of fat synthesis. Inhibition of SREBP-1C could inhibit the transcription of ACC and FAS, thus reducing the synthesis of liver fat [34]. In this experiment, compared with HFD group, after *L. paracasei* JY062 intervention, the contents of AdipoQ and AMPK increased, while the expression levels of SREBP-1c, ACC, and FAS decreased significantly, which was consistent with previous study. These results indicated that energy restriction could activate the APN-AMPK pathway, inhibit the expression of SREBP-1c, down-regulate its target genes FAS and ACC, and reduce lipid synthesis. Moreover, AMPK activated the downstream factors GLUT-4 and PGC-1α. GLUT-4, as one of the markers of insulin resistance, was involved in glucose metabolism. After activation of AMPK, GLUT4 can be translocated to the plasma membrane to increase GLUT4 transcription and improve the uptake and utilization of glucose by the liver through an insulin-independent pathway [35]. PGC-1α is a key signal molecule that regulated mitochondrial biosynthesis, promoted nuclear coding mitochondrial gene transcription, induced mitochondrial synthesis, and accelerated mechanical energy metabolism. Meanwhile, PGC-1α also leads to GLUT4 synthesis. In this experiment, compared with the HFD group, after *L. paracasei* JY062 intervention, the contents of GLUT-4 and PGC-1α increased, which was consistent with previous studies. In conclusion, *L. paracasei* JY062 could improve glucose and lipid metabolism through the APN-AMPK pathway.

The gut microbiota could regulate the body’s glycolipids by participating in the body’s metabolism. Studies have shown that a long-term high-glucose-fat diet could cause disturbances in the intestinal environment, altering the composition, structure, and diversity of the microbiota [36]. In this study, the Alpha diversity index of the mice in the HFD group decreased, indicating that a high-glucose-fat diet could reduce the richness and diversity of gut microbes, which, in turn, could lead to an imbalance in the gut microbiota [27]. After *L. paracasei* JY062 intervention, it could reduce the abundance and diversity of gut microbes. Consistent with Zheng’s findings, the diversity of gut microbiota was restored and the richness of the microbiota increased [37]. The results of PCoA analysis in Beta diversity showed that the structure of the gut microbiota in HFD mice was significantly different from those in the N group and *L. paracasei* JY062 intervention groups, indicating that gut microbiota imbalance could lead to structure of gut microbes and composition changes, and that the intervention of *L. paracasei* JY062 has a certain recovery effect.

Studies have shown that a high-fat diet could induce an increase in the abundance of Firmicutes and Proteobacteria in mice feces and a decrease in the abundance of Bacteroidetes. Among them, Firmicutes could effectively absorb energy from food and cause the body to produce obesity symptoms. Proteobacteria includes many pathogenic bacteria, which induce inflammation, while the decrease in the proportion of Bacteroidetes can lead to metabolic disorders and cause metabolic diseases [38,39]. The results showed that the abundance of Firmicutes at the phylum level was increased and the abundance of Bacteroidetes was decreased in the HFD group, while the LPL group, the results indicate increased Firmicutes after gavage with *L. paracasei* JY062, which might be the result of Lactobacillus at the phylum level. The increase in number can be verified in the results at the genus level in this study, while the abundance of Firmicutes decreased in the LPM and LPH groups. Moreover, the abundance of Bacteroidetes and Proteobacteria in the three dose groups was lower than in the HFD group. Overall, the ratio of upper Bacteroidetes to Firmicutes showed an increased trend, indicating that the intervention of *L. paracasei* JY062 could reduce harmful bacteria and increase the abundance of beneficial bacteria. At the genus level, the HFD group was dominated by Dubosiella, an uncultured Muribaculaceae, and Dubosiella can produce lipopolysaccharide and cause inflammation, and its abundance is correlated with the occurrence of GLMD [40]. Muribaculaceae, one of the dominant bacteria, breaks down carbohydrates. In this study, the abundance of Muribaculaceae in the HFD group was lower than in the N group but not significantly, which may be due to the difference in dietary fiber intake between the two groups of mice. The experimental results showed that the intake of cellulose in the HFD group was higher than in the N group, and cellulose promoted the proliferation of Muribaculaceae. In addition, the abundance of beneficial bacteria Lactobacillus, Akkermansia, and Bifidobacterium significantly increased after *L. paracasei* JY062 was administered orally. Lactobacillus and Bifidobacterium are the most commonly used probiotic strains in clinical trials. Among them, Lactobacillus could change lipid and carbohydrate synthesis pathways and regulate glycolipid metabolism, and Bifidobacterium is a SCFAs-producing bacteria, which can improve the metabolic activity of gut microbiota [41]. Studies have shown that the mixture of Lactobacillus and Bifidobacterium could inhibit the harm of a high-fat diet to the human body through butyric acid, improve the barrier function of the gastrointestinal tract, and inhibit the expression of pro-inflammatory factors [42]. However, there is a direct relationship between Akkermansia and T2DM and other glucolipid metabolic diseases [41]. Compared with obese people, Akkermansia is more abundant in healthy people, and after supplementing Akkermansia, blood glucose and lipid indexes were significantly decreased and alleviated [42].

SCFAs are carbon chain 1~6 organic fatty acids that are generated from undigested starch and fiber polysaccharides through glycolysis by anaerobic bacteria [43]. Studies showed that SCFAs played a critical role in the prevention and treatment of obesity and T2DM in both animal model and clinical trials [40]. Acetic acid is the most abundant SCFA in serum, and it can regulate the level of inflammation, resist the invasion of pathogens, and inhibit the accumulation of fat in adipose tissue [44], and propionic acid stimulates leptin to reduce TC level [45]. Butyric acid can promote the release of gastrointestinal peptide hormones such as peptide YY (PYY) and glucagon-like peptide-1 (GLP-1), thus improving obesity and insulin sensitivity induced by a high-fat diet [46]. In addition, butyric acid activates the liver AMPK signaling pathway, which promotes fatty acid oxidation and inhibits gluconeogenesis and fat production [47]. In this experiment, *L. paracasei* JY062 significantly stimulated the production of acetic acid, propionic acid, and butyric acid. *L. paracasei* JY062 could promote the SCFAs, which is consistent with the above report [48].

After the intervention of *L. paracasei* JY062, the gut microbiota composition of GLMD mice was improved and tended to be in a balanced state of flora, and the relative abundance of SCFAs-producing bacteria such as Lactobacillus, Akkermansia, and Bifidobacterium increased, which stimulated the production of SCFAs and promoted the production of adipose tissue. The secretion of adiponectin increases leptin sensitivity, which, in turn, mediates the adipose–insulin axis. After *L. paracasei* JY062 intervention, it could be found that the relative abundance of Bifidobacterium, Muribacalum, and Turicibacter all increased, and the relative abundance of Faecalibaculum decreased. This indicated that *L. paracasei* JY062 could increase the butyrate content by increasing the relative abundance of Bifidobacterium, Muribacalum, and Turicibacter and decreasing the relative abundance of Faecalibaculum. The above results suggest that *L. paracasei* JY062 can increase the content of SCFAs by increasing the relative abundance of bacteria such as Bifidobacterium, Candidatus_Saccharimonas, Muribacalum, and Turicibacter in the intestinal contents of mice, thereby regulating glucolipid metabolism disorders.

## 5. Conclusions

After the intervention of *L. paracasei* JY062, the gut microbiota composition of GLMD mice was improved and tended to be in a balanced state of flora, and the relative abundance of SCFAs-producing bacteria such as Lactobacillus, Akkermansia, and Bifidobacterium increased and stimulated the production of SCFAs, promoting the production of adipose tissue. On the one hand, adiponectin could bind to AdipoR2 in the liver, activate the AMPK signaling pathway, inhibit the expression of SREBP-1c, promote the expression of GLUT-4, and improve the biochemical indicators of glycolipid; on the other hand, the improvement of leptin sensitivity could improve leptin resistance and better play the role of leptin in controlling food intake, increasing energy consumption, and improving insulin sensitivity, thereby relieving GLMD.

## Figures and Tables

**Table 1:** Primer sequences used for RT-qPCR.

Genes	Primer Sequences	
	Forward (5′–3′)	Reverse (5′–3′)
β-actin	CTACCTCATGAAGATCCTGACC	CACAGCTTCTCTTTGATGTCAC
AdipoQ	CCAATGTACCCATTCGCTTTAC	GAAGTAGTAGAGTCCCGGAATG
Adipor2	CTAATGCTTATGGCTAGCCTCT	AGAGTGAAACCAGATGTCACAT
AMPK	AACCTGAGAACGTCCTGCTTGATG	TGACTTCTGGTGCGGCATAATTGG
SREBP-1c	GCTACCGGTCTTCTATCAATGA	CGCAAGACAGCAGATTTATTCA
ACC	AGATCGCCTCCACCATCGTAGC	CTGTCCTCCGTCCACTCCACTG
FAS	CAAGTGCAAACCAGACTTCTAC	GCACTTTCTTTTCCGGTACTTT
GLUT-4	GCTGGTGTGGTCAATACGGTCTTC	CCAAGCAGGAGGACGGCAAATAG
PGC-1α	ATGTGTCGCCTTCTTGCTCTTCC	CTCCCGCTTCTCGTGCTCTTTG

**Table 2:** Effects of *L. paracasei* JY062 on blood lipid index of mice.

Group	TC (mmol/L)	TG (mmol/L)	LDL-C (mmol/L)	HDL-C (mmol/L)
N	2.73 ± 0.18 ^c^	0.97 ± 0.07 ^c^	0.77 ± 0.09 ^c^	3.70 ± 0.67 ^a^
HFD	5.71 ± 1.07 ^a^	1.79 ± 0.07 ^a^	1.60 ± 0.18 ^a^	1.84 ± 0.55 ^c^
LPL	4.20 ± 0.39 ^b^	1.30 ± 0.05 ^b^	1.18 ± 0.09 ^b^	2.69 ± 0.18 ^b^
LPM	3.08 ± 0.63 ^bc^	1.00 ± 0.06 ^c^	0.88 ± 0.16 ^c^	2.98 ± 0.08 ^ab^
LPH	3.42 ± 0.53 ^bc^	1.03 ± 0.20 ^c^	0.97 ± 0.16 ^bc^	3.78 ± 0.46 ^a^

Different letters denote the significant difference between different groups of mice (*p* < 0.05).

## Data Availability

Data available on request due to restrictions eg privacy or ethical. The data presented in this study are available on request from the corresponding author. The data are not publicly available due to principle of confidentiality.
